# Comparative effects of flywheel and traditional resistance training on reactive strength and multidirectional COD in elite badminton players

**DOI:** 10.3389/fphys.2025.1712464

**Published:** 2025-12-18

**Authors:** Shiwen Tan, Zepeng Lu, Shurui Yuan, Zijie Zhang, Yixuan Zou, Meiyi Zhang, Litian Zhang, Shuairan Li, Jin Dai

**Affiliations:** 1 Sports Coaching College, Beijing Sports University, Beijing, China; 2 Physical Education Center, Xi’an Jiaotong Liverpool University, Suzhou, China; 3 School of Information Engineering, Liaoning Vocational University of Technology, Jinzhou, Liaoning, China; 4 Physical Education Subject Group, Guangzhou Baiyun District Guangda Fuzhong Experimental Middle School, Guangzhou, China

**Keywords:** change-of-directionability, elite badminton athletes, flywheel resistance training, neuromuscular adaptations, reactive strength

## Abstract

**Purpose:**

This study compared the effects of flywheel resistance training (FRT) versus traditional resistance training (TRT) on reactive strength, dynamic balance, and sport-specific change-of-direction (COD) ability in elite badminton players.

**Methods:**

Twenty-four athletes (mean age: 21.2 ± 2.2 years) were randomly allocated to an FRT group (n = 12) or a TRT group (n = 12). Over 6 weeks, both groups performed twice-weekly sessions of squats, deadlifts, and lunges at a rating of perceived exertion ≈8. Performance was assessed before and after the intervention using the SEMO agility test, modified 5–10-5 COD test, 10-m sprint, reactive strength index (RSI), and Y-Balance test. Data were analyzed with a two-way mixed ANOVA (p < 0.05).

**Results:**

Mixed ANOVA revealed significant main effects of time for the SEMO test, modified 5–10-5 COD test, and 10-m sprint (all *p* < 0.001, η^2^ = 0.56–0.79), indicating performance improvements at post-test in both training groups. No significant group × time interactions were observed for these variables (*p* = 0.07–0.23). Within-group effect sizes for these speed and COD measures were large in the FRT group (SEMO: Cohen’s d = 1.01; 5–10-5: d = 1.21; 10-m sprint: d = 1.27) and ranged from small to large in the TRT group (d = 0.40–0.87). For RSI, both the main effect of time and the group × time interaction were significant (*p* < 0.001, η^2^ = 0.90 and 0.66, respectively). Within-group effect sizes were large in the FRT group (d = 2.43) and moderate in the TRT group (d = 0.79). Y-Balance Test improved over time for both dominant and non-dominant limbs (*p* < 0.001, η^2^ = 0.53–0.57), with no significant group × time interaction (*p* > 0.75).

**Conclusion:**

These findings suggest that FRT may provide additional advantages over TRT in enhancing reactive strength and COD ability, likely due to eccentric overload-induced neuromuscular adaptations. However, both training modalities were similarly effective in improving dynamic balance. These findings have practical implications for coaches: incorporating twice-weekly FRT into badminton-specific conditioning may optimize deceleration mechanics and multidirectional COD and improve transfer of strength gains to on-court performance.

## 1 Introduction

Badminton is a high-intensity, intermittent sport that requires rapid directional changes and explosive lower-limb power. During match play, players perform approximately 8–10 directional changes per minute, demanding exceptional COD ability for optimal performance (Cabello Manrique and González-Badillo, 2003; Phomsoupha and Laffaye, 2015; Shariff et al., 2009; Hong et al., 2014; Wang et al., 2019). Efficient COD movements not only enhance stroke accuracy and timing but also enable effective positioning and recovery between rallies (Wei et al., 2008). Eccentric strength, lower-limb power, and dynamic balance are key determinants of performance, enabling rapid deceleration, efficient reacceleration, and precise technical execution (Dos’santos et al., 2021; Dosʼsantos et al., 2017; Lu et al., 2022; Zhou et al., 2022).

Agility is a multidimensional ability involving rapid whole-body changes in speed or direction in response to external stimuli. It integrates perceptual-cognitive processing, neuromuscular coordination, and biomechanical control. Reactive strength refers to the neuromuscular system’s ability to efficiently utilize the stretch-shortening cycle by quickly transitioning from eccentric to concentric muscle action; it is commonly quantified using the RSI derived from jump performance. Recent studies emphasize that agility and speed are strongly influenced by intrapersonal movement characteristics such as functional movement quality, dynamic balance, and coordination. For instance, Koźlenia et al. linked movement pattern quality to speed–agility performance in team-sport athletes (Koźlenia et al., 2020), while Alexe et al. and Grabara and Bieniec found dynamic balance and movement efficiency to be key predictors of agility across football and ice hockey players (Alexe et al., 2024; Grabara and Bieniec, 2024).

Given the central role of COD and agility in badminton, researchers have proposed diverse training approaches, including ladder drills, pre-planned COD tasks, perceptual-cognitive agility training, plyometrics, and resistance-based strength programs (Guo et al., 2021; Wang et al., 2022; Friebe et al., 2024). Among these, resistance training is particularly relevant for badminton, as it enhances eccentric strength and stretch-shortening cycle function—both essential for repeated braking and acceleration movements. Traditional percentage-based training has been reported to improve lower-limb strength (Khazaei et al., 2023; Kibele and Behm, 2009), balance (Khazaei et al., 2023; Kibele and Behm, 2009; Wang et al., 2023), and COD performance (Silva et al., 2015). Eccentric strength of the hip extensors, quadriceps, and gastrocnemius is crucial for effective deceleration (Smith et al., 2009; Mccurdy et al., 2014), which facilitates subsequent acceleration and contributes to overall athletic performance (Dos’santos et al., 2021; Dos’santos et al., 2018; Chaabene et al., 2018). However, the recent meta-analysis results have confirmed that in percentage-based training, the application of centrifugal force is limited by the centripetal force capability, usually only reaching 40%–50% of the maximum centrifugal force (Nuzzo et al., 2023), which may not be sufficient to meet the movement requirements of rapid direction changes in badminton.

FRT has recently gained substantial attention for its capacity to impose both enhanced eccentric and concentric loading through inertial resistance systems, thereby providing distinct neuromuscular stimuli compared with TRT. Early experimental research indicated that FRT can produce eccentric loads around 120%–130% of concentric effort (Norrbrand et al., 2008), recent consensus highlights that true eccentric overload is not universally achieved and depends on factors such as exercise selection, inertia magnitude, braking technique, and participant experience (Beato et al., 2024). Advances in flywheel technology have further enhanced the ability to monitor these mechanical outputs with precision, enabling more controlled and quantifiable eccentric stimuli. Moreover, long-term interventions and systematic reviews indicate that FRT promotes superior adaptations in lower-limb strength, sprint ability, and change-of-direction performance compared with TRT, primarily through greater eccentric force generation and improved neuromuscular efficiency (Allen et al., 2023). Collectively, these findings underscore the sustained efficacy of FRT as a reliable and quantifiable training method for optimizing sport-specific performance through targeted eccentric and concentric loading.

Although FRT has been previously explored as a method to enhance COD performance, most existing studies have focused on short-term interventions, non-elite populations, or isolated performance variables such as sprint speed or jump height. Evidence on its long-term efficacy in improving reactive strength, dynamic balance, and badminton-specific COD ability among elite athletes remains limited and inconclusive. Compared with field-based sports, badminton requires frequent, high-intensity directional changes within a confined space, demanding exceptional eccentric control, postural stability, and rapid neuromuscular transitions through the stretch-shortening cycle. Therefore, directly comparing FRT with TRT under badminton-specific conditions provides novel insights into sport-specific neuromuscular adaptations. Building on this evidence, the present study focuses on three primary determinants of agility—linear speed, dynamic balance, and reactive strength—assessed using the 10-m sprint, Y-Balance Test, and RSI, respectively, in combination with SEMO and modified 5–10-5 COD tests. We hypothesized that, owing to its continuous inertial resistance and greater eccentric loading capacity, FRT would elicit superior improvements in reactive strength and COD performance compared with TRT.

## 2 Methods

### 2.1 Participants

A mixed-design ANOVA was conducted. The required sample size was estimated using G*Power 3.1, assuming a medium effect size (f = 0.25), α = 0.05, and statistical power (1-β) = 0.80, yielding a minimum of 24 participants. This estimation was based on prior evidence reporting moderate-to-large improvements in change-of-direction performance following resistance-based interventions, including a meta-analysis showing large effects (SMD ≈ −0.82) (Chaabene et al., 2020), a narrative review confirming consistent COD gains (Nygaard et al., 2019), and long-term strength training studies demonstrating significant COD enhancements in trained athletes (Keiner et al., 2014). Elite badminton athletes were included if they: (1) had competed at provincial finals or national youth quarterfinals or above; (2) possessed a back-squat 1RM ≥ 1.5 times body mass; and (3) could complete the 8-week program. Exclusion criteria included any lower-limb injury within the previous 3 months. All participants provided written informed consent after being informed of the purpose, risks, and benefits. They were instructed to maintain their habitual diet and avoid caffeinated beverages throughout. The study was approved by the Institutional Ethics Committee of Beijing Sport University (2023280H) and conducted in accordance with the Declaration of Helsinki.

### 2.2 Experimental approach to the problem

This 6-week randomized controlled trial compared FRT with TRT. Participants were randomly assigned to either group. Participants were randomly assigned to either group using a computer-generated randomization table automatically created in Microsoft Excel, based on unique identification numbers assigned before the intervention. Both groups performed two resistance training sessions per week, scheduled 48 h apart, in conjunction with their regular badminton-specific technical–tactical sessions (five times per week, approximately 2 h each). The resistance training was integrated into the athletes’ ongoing training routine rather than performed in isolation. This design ensured ecological validity while minimizing interference between strength and sport-specific training. Performance was assessed pre- and post-intervention (within 72 h). To minimize fatigue, high-intensity exercise was prohibited ≥24 h before testing. All tests were conducted under controlled conditions (20 °C–25 °C; 40%–50% humidity). Standardized instructions and 1-2 familiarization trials were provided before each test. After a standardized warm-up, maximal effort was required. Rest intervals (5–10 min) and consistent timing enhanced reliability. A 2-week familiarization period preceded the intervention.

The FRT group performed lower-limb exercises using a flywheel inertial device (D Full, Italy), including the squat, deadlift, and lunge, as outlined in Table 1. Each session comprised four sets per exercise at an exertion level corresponding to 8 on the Borg CR10 scale (Hackett et al., 2012), executed at maximal concentric velocity with 3-min rest intervals between sets. For the FRT group, inertia was progressively increased by combining plates (Pro, Large, Medium, Small) to maintain the target exertion level, while in the TRT group, external loads were set at approximately 80% 1RM and adjusted if perceived exertion deviated by more than one point (Yuan et al., 2025). All training sessions were supervised by certified strength and conditioning specialists to ensure technical accuracy, controlled movement tempo, and adherence to prescribed intensity.

**TABLE 1:** Training plans for FRT and TRT groups.

Training cycle	Group	Training content	Load plates	Load	Rest interval
Weeks 1–2	FRT	Flywheel squat	P + L + M, L	4 sets (8 RPE)	3 min
		Flywheel deadlift	P, L		
	TRT	Barbell squat	80% 1RM	4 sets (8 RPE)	3 min
		Barbell deadlift			
Weeks 3–4	FRT	Flywheel lunge	P + L + M, L	4 sets (8 RPE)	3 min
		Flywheel deadlift	P, L		
	TRT	Barbell lunge	80% 1RM	4 sets (8 RPE)	3 min
		Barbell deadlift			
Weeks 5–6	FRT	Flywheel squat	P + L + M, L	4 sets (8 RPE)	3 min
		Flywheel lunge	P + L + M, L		
	TRT	Barbell squat	80% 1RM	4 sets (8 RPE)	3 min
		Barbell lunge			

Abbreviations: P, pro plate; L, large plate; M, medium plate; S, small plate.

**FIGURE 1:**
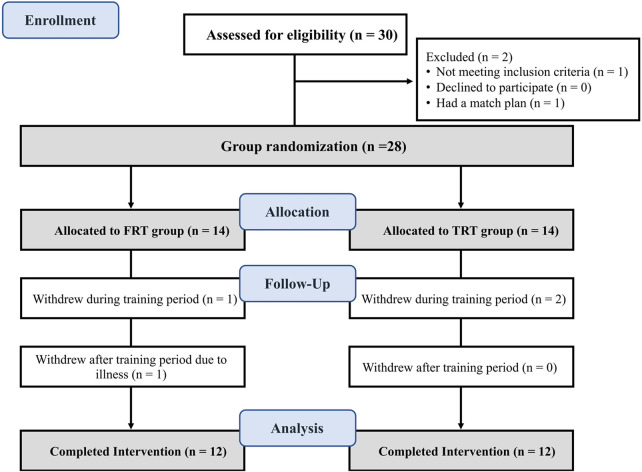
Flow diagram of the participants.

Initially, 28 participants were enrolled. Four withdrew due to competition conflicts, resulting in 24 athletes included in the final analysis (Figure 1). Baseline characteristics showed no significant differences between groups (*P* > 0.05, Table 2).

**TABLE 2:** The demographic characteristics of the participants.

Group	Age (years)	Height (cm)	Weight (kg)	Training experience (years)
FRT (n = 12)	20.64 ± 2.24	175.27 ± 8.38	69.91 ± 9.22	11.27 ± 2.00
TRT (n = 12)	21.19 ± 2.04	178.70 ± 4.76	69.80 ± 8.24	11.95 ± 2.42

### 2.3 Test program

#### 2.3.1 SEMO test

**FIGURE 2:**
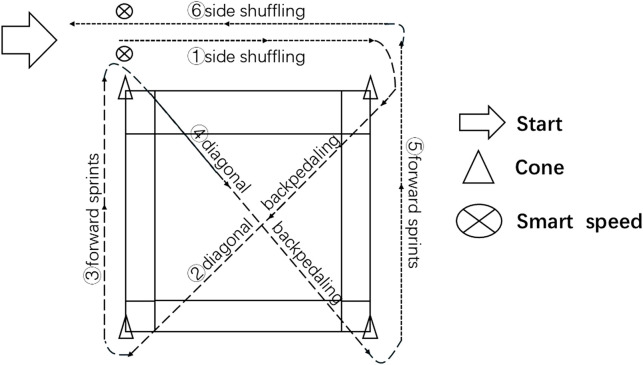
SEMO test.

The SEMO test (Guo et al., 2021) was administered on a regulation badminton court. Four cones were placed at the intersections of the front service line and doubles sideline, and the back boundary and doubles sideline (Figure 2). Starting 1 m outside one front intersection, performance time was recorded via a photoelectric system (Smart Speed™, Australia; ±0.01 s accuracy). Beginning facing away from the court, participants moved laterally upon the start signal, weaving around each cone before returning to the start. All participants adhered strictly to the movement pattern. After three maximal trials with 5-min recovery intervals, the best time was used for analysis.

#### 2.3.2 Modified 5–10-5 COD test

**FIGURE 3:**
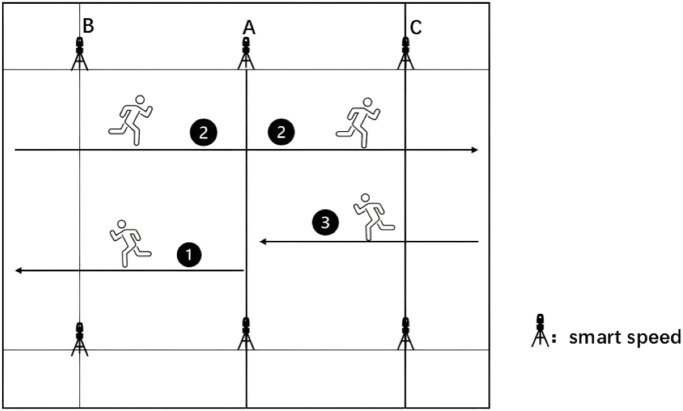
Modified 5–10-5 COD test.

A modified 5–10-5 COD test (Guo et al., 2021) was implemented with distances calibrated to a standard badminton court width (6.10 m) to enhance ecological validity. Six cones were positioned at point A (intersection of front service line and centerline) and points B and C (intersections of front service line and doubles sidelines; see Figure 3). Smart Speed timing gates recorded performance. Starting facing the net at point A, participants turned on cue, sprinted to point B, touched the cone with hand and foot, repeated this at point C, and then sprinted back to point A. Three trials with 4-min recovery intervals were completed. The fastest valid trial was used for statistical analysis.

#### 2.3.3 10-m Linear Sprint Test

**FIGURE 4:**
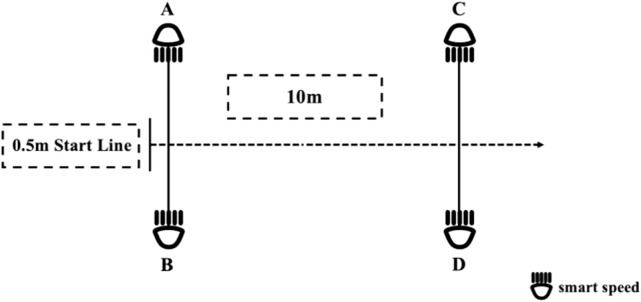
10-m linear sprint test.

A 10-m sprint test was used to assess maximal acceleration. Timing gates (Smart Speed) were positioned at the start and finish lines, marked by cones. After two warm-up sprints, participants started from a three-point stance and sprinted maximally to the finish. Two trials were performed separated by 4-min rest periods. The best time was retained for analysis. The testing configuration is shown in Figure 4.

#### 2.3.4 RSI test

RSI was used to assess stretch-shortening cycle (SSC) utilization (Guo et al., 2021). Measurements were obtained using a force platform (Kistler, Switzerland; 1,000 Hz) with a 30-cm platform. From an upright position on the platform, participants dropped without a countermovement to ensure zero initial velocity. Landings were confined to the force plate center (<10 cm). Upon landing, participants immediately performed a maximal vertical jump. Force-time data were used to compute contact time, flight time, jump height, and RSI, calculated as:

$$RSI=\frac{Jump\;Height}{Ground\;contact\;time}$$

Three maximal jumps were performed with 90-s rest intervals. The best trial was used for analysis, with results recorded to 0.01 s.

#### 2.3.5 Y-balance test

Lower limb length was measured (anterior superior iliac spine to medial malleolus; ±0.5 cm) prior to Y-Balance testing (Guo et al., 2021), Participants performed the test barefoot using a Y-Balance™ kit (Functional Movement Systems, United States of America). While maintaining unilateral stance on the dominant leg, they performed maximal reaches in anterior, posteromedial, and posterolateral directions with the non-stance limb, returning to the start between attempts. The maximum reach distance in each direction (recorded to 0.1 cm) was determined from three trials. Overall dynamic balance performance was quantified using a normalized composite score, calculated as follows:

$$Composite\;Score=\frac{\left(Anterior+Posteromedial+Posterolateral\right)}{3\times Right\;Limb\;Length}\times 100$$

### 2.4 Statistical analysis

Statistical analyses were performed using IBM SPSS Statistics (v 27.0). Continuous data are expressed as mean ± standard deviation. A two-way mixed-design ANOVA evaluated main and interaction effects of group (between-subjects: FRT vs. TRT) and time (within-subjects: pre-vs. post-intervention) on dependent variables: (i) Change-of-direction: SEMO, modified 5–10-5 tests; (ii) Acceleration: 10-m sprint; (iii) Reactive strength: RSI; (iv) Dynamic balance: Y-Balance normalized composite scores (dominant/non - dominant limbs). Mauchly’s test assessed sphericity assumption violations; Greenhouse-Geisser corrections adjusted degrees of freedom where ε < 0.75. Effect sizes for interactions were quantified via partial *η* ^2^ (small: <0.06; medium: 0.06–0.14; large: ≥0.14) (Cohen, 1988). Bonferroni-adjusted pairwise comparisons followed significant main/interaction effects (*p* < 0.05). Magnitude thresholds for Cohen’s d (pairwise contrasts) were: trivial (<0.2), small (0.2–0.6), medium (0.6–1.2), large (1.2–2.0), very large (>2.0). Statistical significance was set at *p* < 0.05.

## 3 Results

**FIGURE 5:**
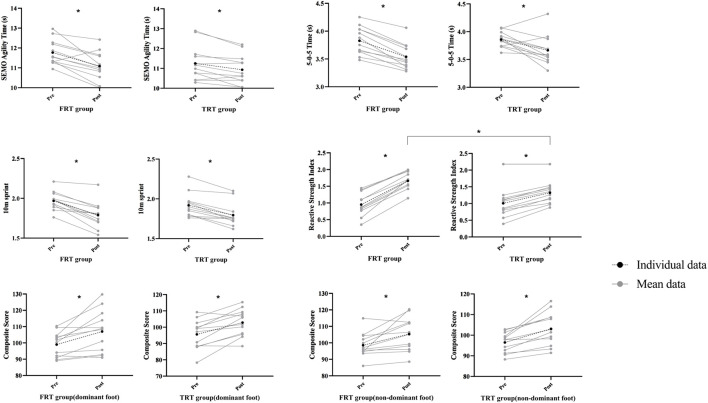
Individual and mean values for enhancing reactive strength and COD related tests during before and after FRT and TRT. Note: *p< 0.05.

Table 3 summarizes the descriptive statistics and mixed-design ANOVA outcomes for primary dependent variables: SEMO agility, modified 5–10-5 COD, 10-m sprint, RSI, and bilateral Y-Balance normalized composite scores. Crucially, baseline between-group equivalence was confirmed for all measures (*p* > 0.05). Figure 5 depicts within- and between-group ESs for comparisons of test results.

**TABLE 3:** The assessment results for FRT group and TRT group before and after the 6-week training.

	FRT (n = 12)			TRT (n = 12)			Time		Group*Time	
	Pre	Post	ES	Pre	Post	ES	*p*	*η* ^2^	*p*	*η* ^2^
SEMO test (s)	11.77 ± 0.63	11.09 ± 0.72^a^	1.01	11.25 ± 0.88	10.93 ± 0.73^a^	0.40	<0.001	0.560	0.069	0.143
5–10-5 COD test (s)	3.83 ± 0.25	3.54 ± 0.23^a^	1.21	3.86 ± 0.14	3.67 ± 0.27^a^	0.88	<0.001	0.647	0.232	0.064
10-M sprint	1.97 ± 0.12	1.79 ± 0.16^a^	1.27	1.92 ± 0.15	1.79 ± 0.15^a^	0.87	<0.001	0.789	0.118	0.108
RSI	0.95 ± 0.33	1.66 ± 0.25^a^ ^,^ ^b^	2.43	1.00 ± 0.45	1.32 ± 0.35^a^	0.79	<0.001	0.931	<0.001	0.663
YBT (dominant foot)	99.03 ± 7.60	107.04 ± 12.89^a^	0.76	95.61 ± 8.96	102.83 ± 8.16^a^	0.84	<0.001	0.565	0.783	0.004
YBT (non-dominant foot)	98.62 ± 7.27	105.38 ± 10.18^a^	0.76	96.47 ± 4.92	103.09 ± 8.12^a^	0.69	<0.001	0.530	0.958	0.000

^a^ Statistically significant difference between pre- and post-test, *p* < 0.05.

^b^ Between-group differences (FRT, vs. TRT) at post-intervention between FRT, group and TRT, group, *p* < 0.05.

### 3.1 SEMO test

A significant main effect of time was observed for SEMO test performance (F_(1, 22)_ = 28.056, *p* < 0.001, *η* ^2^ = 0.560). However, the interaction effect between group and time was not statistically significant (F_(1, 22)_ = 3.659, *p* = 0.069, *η* ^2^ = 0.143). Post hoc analyses indicated significant within-group improvements for both FRT (*p* < 0.001, Cohen’s d = 1.01) and TRT (*p* = 0.026, Cohen’s d = 0.40) relative to baseline. The FRT group showed a 153% greater effect size magnitude versus TRT.

### 3.2 Modified 5–10-5 COD test

A significant main effect of time was observed for modified 5–10-5 test performance (F_(1, 22)_ = 40.274, *p* < 0.01, *η* ^2^ = 0.647). However, the group × time interaction effect was not statistically significant (F_(1, 22)_ = 1.512, *p* = 0.232, *η* ^2^ = 0.064). Post hoc tests confirmed significant within-group improvements from baseline for FRT (*p* < 0.001, Cohen’s d = 1.21) and TRT (*p* = 0.003, Cohen’s d = 0.88).

### 3.3 10-m sprint test

A significant main effect of time was showed for 10-m sprint performance (F_(1, 22)_ = 82.420, *p* < 0.0 1, *η* ^2^ = 0.789). However, the group × time interaction effect was not statistically significant (F_(1, 22)_ = 2.653, *p* = 0.118, *η* ^2^ = 0.108). Post hoc analyses confirmed significant within-group improvements for FRT (*p* < 0.001, Cohen’s d = 1.27) and TRT (*p* < 0.001, Cohen’s d = 0.87). Notably, FRT yielded a 46% greater effect magnitude compared to TRT.

### 3.4 Reactive strength index test

A significant main effect of time was observed for RSI scores (F_(1, 22)_ = 295.896, *p* < 0.001, *η* ^2^ = 0.931), along with a significant group × time interaction effect (F_(1, 22)_ = 43.292, *p* < 0.001, *η* ^2^ = 0.663). Simple effects analyses confirmed post-intervention RSI improvements in both FRT (*p* < 0.001, Cohen’s d = 2.43) and TRT (*p* < 0.001, Cohen’s d = 0.79). Crucially, FRT showed greater RSI enhancement versus TRT at post-test (*p* = 0.012).

### 3.5 Y-Balance Test

For the dominant limb Y-Balance test, a significant main effect of time was observed (F_(1, 22)_ = 28.605, *p* < 0.001, *η* ^2^ = 0.565), with no group × time interaction (F_(1, 22)_ = 0.078, *p* = 0.783, *η* ^2^ = 0.004). Post hoc analyses indicated significant within-group improvements for both FRT (*p* < 0.001, Cohen’s d = 0.76) and TRT (*p* = 0.002, Cohen’s d = 0.84), demonstrating comparable intervention efficacy (*p* = 0.349 for between-group difference).

Correspondingly, non-dominant limb performance showed a significant time effect (F_(1, 22)_ = 24.822, *p* < 0.001, *η* ^2^ = 0.530) and no interaction (F_(1, 22)_ = 0.003, *p* = 0.958, *η* ^2^ < 0.001). Both groups improved significantly (FRT: *p* = 0.002, Cohen’s d = 0.76; TRT: *p* = 0.002, Cohen’s d = 0.69), with no between-group advantage (*p* = 0.550).

## 4 Discussion

The present findings indicate that 6 weeks of FRT elicit superior improvements in reactive strength compared with TRT. Despite the absence of statistically significant differences in COD ability, dynamic balance, or 10-m sprint performance, the greater effect sizes observed in the FRT group suggest meaningful practical advantages.

The SEMO and modified 5–10-5 tests are designed to mimic essential movement patterns in badminton, including rapid COD, deceleration, and reorientation-skills that are frequently utilized during competitive play. Effective performance in COD tasks is critically linked to an athlete’s acceleration-deceleration ability, dynamic stability, and explosive power of the lower-limbs. In badminton, explosive strength enables quick transitions, whereas dynamic balance helps maintain postural stability and manage inertia, allowing smoother, more controlled execution of sport-specific actions.

Although no significant between-group differences were observed in COD performance or dynamic balance, the larger effect sizes favoring the FRT group suggest that this modality may contribute to enhanced eccentric deceleration control and postural stability, which are key determinants of COD ability (Yuan et al., 2025; Morencos et al., 2022). During FRT, the lower limbs perform dynamic braking under high tension, thereby strengthening the proprioceptive system and postural control (Da Silva Araújo et al., 2025). These results align with emerging evidence that eccentric overload inherent to FRT promotes enhanced neuromuscular adaptation, modulates tendon stiffness, and optimizes the SSC, thereby providing a more potent stimulus for performance development than conventional resistance methods. Zhang et al. (2019) reported that eccentric training improved COD performance in tennis players, and Núñez et al. (2018) observed enhanced on-court performance following eccentric overload training.

The superior improvement with FRT is likely attributable to its inertial-overload mechanism (Coratella et al., 2019), which imposes maximal eccentric effort to decelerate the flywheel immediately before the concentric phase. The continuous tension during the eccentric - concentric transition closely mirrors SSC demands in badminton-specific maneuvers, thereby enhancing neuromuscular efficiency and potentiating the SSC (Coratella et al., 2019; Beato et al., 2021). In contrast, TRT relies on gravity-dependent loading and does not provide comparable eccentric overload or inertial stimuli (Yuan et al., 2025; Moritani et al., 1987), resulting in suboptimal adaptation for SSC performance. The neuromuscular activation elicited by FRT more closely simulates the deceleration and redirection tasks encountered in badminton, which likely explains its advantage in enhancing reactive strength. Moreover, the inherently unstable inertial characteristics of the flywheel device require continuous movement-to-movement adjustments, promoting improvements in sensorimotor coordination (Beato and Dello Iacono, 2020).

Dynamic stability is essential in badminton for maintaining postural control during rapid, multidirectional movements. Improved balance supports trunk - pelvis coordination and limb control during unstable phases, thereby reducing technical errors and enhancing performance. These results are consistent with Kean et al. (2006), who reported a 10% improvement in jump performance after balance training, attributed to improved control of the center of mass during landing. Such adaptations help minimize excessive motion and improve force transmission. Furthermore, inadequate neuromuscular control and poor landing mechanics are associated with an increased risk of anterior cruciate ligament (ACL) injury (Harper et al., 2019). Eccentric and multiplanar loading strategies, as utilized in FRT, actively enhance neuromuscular control and joint stability, thereby supporting injury prevention and improving performance outcomes.

The improvement in the RSI primarily reflects enhanced efficiency during the eccentric–concentric transition phase. FRT provides a progressively increasing load during the eccentric phase, compelling athletes to generate higher eccentric torque and braking forces during deceleration (Smajla et al., 2022). This eccentric overload stimulus promotes range of neuromuscular adaptations, including greater α-motoneuron recruitment and improved synaptic transmission efficiency, increased high-threshold recruitment of fast-twitch (Type IIx) muscle fibers, and enhanced tendon stiffness accompanied by improved energy storage–release capacity (Beato and Dello Iacono, 2020; Jarosz et al., 2023). Collectively, these physiological adaptations augment reactive muscular output and result in significantly greater RSI improvements after 6 weeks of training compared with TRT. In contrast, the constant-load nature of TRT primarily facilitates hypertrophic adaptations, while providing insufficient stimulus for neural drive enhancement and eccentric control.

## 5 Conclusion

FRT showed superior efficacy compared with TRT in improving reactive strength and COD performance in elite badminton players. This advantage is likely attributable to the eccentric overload and multiplanar loading inherent in FRT, which enhance SSC function and sport-specific agility. Both training modalities produced comparable improvements in dynamic balance, indicating that either approach can effectively enhance postural stability. These findings support integration of FRT into badminton-specific conditioning programs, with two weekly sessions recommended to optimize reactive strength and COD performance.

## Data Availability

The original contributions presented in the study are included in the article/supplementary material, further inquiries can be directed to the corresponding author.
